# Main Allelochemicals from the Rhizosphere Soil of *Saussurea lappa* (Decne.) Sch. Bip. and Their Effects on Plants’ Antioxidase Systems

**DOI:** 10.3390/molecules23102506

**Published:** 2018-09-30

**Authors:** Jingkun Liu, Min Xie, Xiuzhuang Li, Hui Jin, Xiaoyan Yang, Zhiqiang Yan, Anxiang Su, Bo Qin

**Affiliations:** 1 CAS Key Laboratory of Chemistry of Northwestern Plant Resources and Key Laboratory for Natural Medicine of Gansu Province, Lanzhou Institute of Chemical Physics, Chinese Academy of Sciences, Lanzhou 730000, China; liujk918@163.com (J.L.); xiemin0926@126.com (M.X.); comefine@126.com (H.J.); ruoxi020xw@163.com (X.Y.); yanzq@licp.cas.cn (Z.Y.); 2 University of Chinese Academy of Sciences, Beijing 100049, China; 3 Institute for the Control of Agrochemicals, Ministry of Agriculture (ICAMA), Beijing 100125, China; sdinhk@163.com

**Keywords:** *Costus*, allelochemicals, chemical basis, antioxidase system, replant problems

## Abstract

Allelochemicals are the media of allelopathy and form the chemical bases of plant-environment interactions. To determine true allelochemicals and their autotoxic effects, seven compounds were isolated and identified from *in-situ* sampled rhizosphere soil of cultivated *Saussurea lappa*. Of these; costunolide (**2**), dehydrocostus lactone (**3**) and scopoletin (**4**) showed significant inhibition on seedling growth in a concentration-dependent manner. Detection and observation demonstrated that the antioxidase system was found to be affected by these chemicals, resulting in the accumulation of ROS and membrane damage. To investigate their release ways, the compounds were traced back and volumes quantified in rhizosphere soil and plant tissues. This work made clear the chemical bases and their physiological effects on the plants. These chemicals were found to be the secondary metabolites of the plants and included in the rhizosphere soil. The findings identified a potential pathway of plant-plant interactions, which provided theoretical basis to overcoming replanting problems. This research was also useful for exploring ecological effects of allelochemicals in green agriculture.

## 1. Introduction

*Saussurea lappa* (Decne.) Sch. Bip. is an alpine medicine plant frequently used as an anti-inflammatory, digestion aid and liver protection agent in Traditional Chinese Medicine [1]. In cultivation, it is often stunted by replanting obstacles, resulting in serious plant diseases, low production and poor quality [2,3,4]. In previous reports, allelopathy has been increasingly considered to be the main inducement to growth [5,6,7,8]. It is a major pathway of interactions between the plant and environmental factors, including the plant itself, and allelochemicals are the main media of the interactions [9,10,11,12].

According to the release ways of allelochemicals, including root exudates, decomposition of plant residues and leaching, rhizosphere soil should be the enrichment region of allelochemicals and used for research on allelopathy [13,14,15]. In previous research on allelopathy [16,17,18], the tested chemicals were usually culture medium or plant tissue extracts. These samples may not be regarded as a true representation of allelochemicals as not all chemicals in the plants are released into the environment during a normal plant lifecycle. Furthermore, the metabolites in the culture medium are different from those in the soil because they would vary with the growth conditions of the plants. Therefore, it may be more accurate to take rhizosphere soil from naturally cultivated samples.

Plants are constantly exposed to the environment and allelochemicals are their response mechanisms to deal with the stresses caused by biotic or abiotic factors, and even by other individuals of their own population. Moreover, to obtain more resources, plants will release allelochemicals to restrict the growth of other plants around them and consequently residual chemicals can lead to replanting obstacles. The release and accumulation of allelochemicals cause a disequilibrium in soil ecology. Plants or microbes respond to these stresses, and this is a critical aspect of the interactions. These stresses may affect the enzyme activity, cell structure and material or energy metabolism of plants. The antioxidant system is a fundamental physiological system in plants, which responds rapidly to the stresses and influences other physiological activities. In this system, antioxidant enzymes and reactive oxygen species (ROS) [19] are the sensitive and primary signals in the interaction caused by the allelochemicals. In healthy plants, ROS will trigger positive reactions, such as responsive promotion of genes, phytoalexin synthesis and reinforcement of the cell wall to overcome stress [20]. However, excessive ROS will damage the structure, nucleic acids and proteins of the cell when there is disequilibrium between the ROS generation and elimination systems. In response, the antioxidant enzymes with the major function of ROS scavenging will react rapidly to cope with the increase in stresses [21,22,23]. Thus, the ROS level and the enzyme activity are the important elements of the antioxidant defense system when the plants are treated with the allelochemicals. In this work, rhizosphere soil of cultivated *S. lappa* was sampled in situ without any disturbances. Potential allelochemicals from the crude extract were isolated and determined for a first time although they are distributed in the soil at extremely low levels. Isolated compounds, showing obvious activity to the seedlings in the stress response, were used for the investigation on the antioxidant enzyme system of the receptor plant, including superoxide dismutase (SOD), peroxidase (POD) and malondialdehyde (MDA) [19,24,25]. Accordingly, the ROS in the cells were labelled by fluorescein and observed dynamically under a microscope, which showed clearly the status of the cells under the stress of the allelochemicals. Finally, these compounds were traced back and quantified in the soil and plant tissues to investigate the possible ways of the allelochemicals being released into the environment. This work attempted to clarify which compounds in the allelopathy of *S. lappa* exerted effects on the antioxidant system of plants. Furthermore, this study provides a theory and natural material for potential pesticides based on the interaction between plants and environment, which would be an improvement for green agriculture.

## 2. Results and Discussion

### 2.1. Allelochemicals from the Rhizosphere Soil of S. lappa

**Figure 1:**
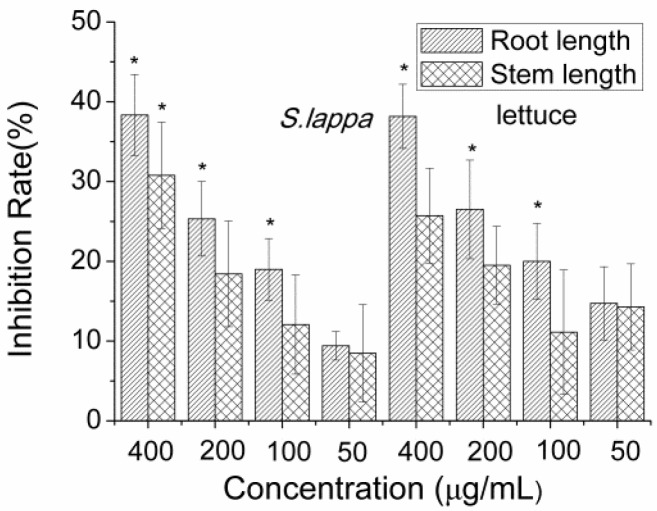
Inhibition of the extract from rhizosphere soil of *S*. *lappa* on the seedlings. Inhibition rates are presented as the mean percentage of the compared to the control. Values significantly lower than the DMSO controls are indicated with one asterisk (Dunnett’s one-sided *t* test, * *p* < 0.05) or two (** *p* < 0.01). Error bars are standard errors of the means, *n* = 3.

Crude extracts from the rhizosphere soil of *S. lappa* showed inhibition both on the same seedlings of *S. lappa* and lettuce in a concentration-dependent manner (Figure 1).

**Figure 2:**
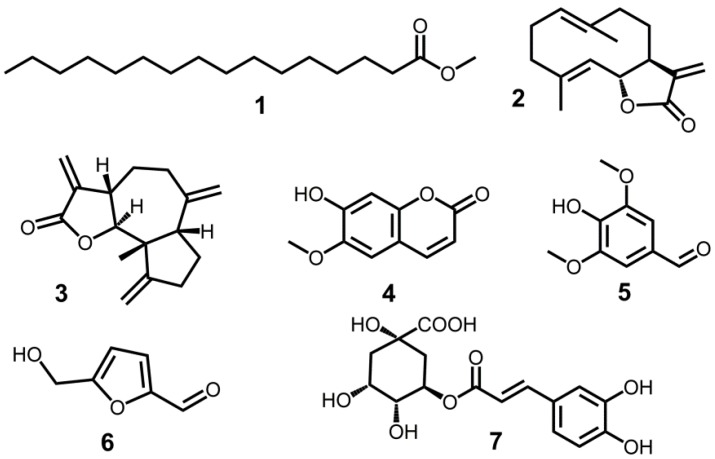
Structures of the compounds isolated and identified from the rhizosphere soil of *S*. *lappa.* (**1**, methyl palmitate; **2**, costunolide; **3,** dehydrocostus lactone; **4**, scopoletin; **5**, syringaldehyde; **6**, 5-hydroxymethylfurfural; **7**, chlorogenic acid).

Bioassay-guided approaches permitted us to separate the more effective fractions in order to obtain its allelophathic compounds. Seven compounds: methyl palmitate (**1**) [26], costunolide (**2**) [27,28,29], dehydrocostus lactone (**3**) [27,29], scopoletin (**4**) [30,31], syringaldehyde (**5**) [32], 5-hydroxymethylfurfural (**6**) [33] and chlorogenic acid (**7**) [34], were isolated and identified. Their structures are displayed in Figure 2.

As reported in [35], five of the seven compounds are definitely secondary metabolites of *S. lappa*, especially compounds **2**, **3** and **4**. They would be synthesized when necessary. The secondary metabolism varies with the growth conditions, including the plant-environment interactions. They were identified as follows.

In ^1^H-NMR spectrum of compound **1**, as reported in the literature [23], the hydrogen atoms with peaks at 3.66, 2.30 and 0.88 ppm characterized the methoxy, red shifted methylene and methyl protons, and no unsaturated carbon was present in the structure. In compound **2**, 6-CH_3_, 10-CH_3_ carbonyl and three ethylenic bonds were the characteristic features, which were identical with the data in [24,25,26]. The chemical shift and coupling constants of the ethylenic bonds in compound **3** were all confirmed by the literature data [24,26]. H-12 (3.96 ppm) showed a triplet peak. Its coupling constant was also significant evidence. There were no saturated hydrocarbon protons in the data of compound **4** or in literatures [27,28], and the characteristic structural features: -OCH_3_, two single unsaturated carbons and two groups of double proton peak, were all identified. The symmetric -OCH_3_ and -H of compound **5** appeared at 3.98 and 7.16 ppm, and the areas of the peaks were also in accordance with the number protons. A -CHO was speculated according to the -H and C=O shifts. All these chemical data are confirmed by the literature [29]. A broad proton peak and a carbon shift of 177.74 ppm proved the active hydrogen and -CHO in compound **6**, respectively. The carbons at 110.02, 122.95, 152.35 and 160.71 ppm were in accordance with the tetrahydrofuran in the reported structure [30]. A benzene ring, -C=C and -OC=O- in compound **7** could be verified by comparison with the literature [31], and the chemical shifts and coupling coefficients of the protons were all identical. A cyclohexane ring could be speculated according to the ^13^C-NMR. The mass spectrum and ^1^H-NMR verified the substituents. All these speculated structures were further conformed by the high-solution mass spectral data and literature reports.

### 2.2. Activity of the Purified Compounds on the Seedling Growth of S. lappa and Lettuce

**Figure 3:**
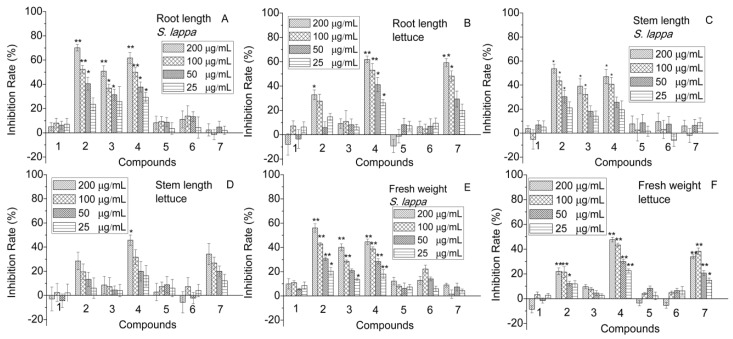
Inhibitions of the purified compounds (**1–7**) on the seedlings. Inhibition rates are presented as the mean percentage of the compared to the control. Values significantly lower than the DMSO controls are indicated with one asterisk (Dunnett’s one-sided *t* test, * *p* < 0.05) or two (** *p* < 0.01). Error bars are standard errors of the means, *n* = 3. ((**A**) root length, *S. lappa*; (**B**) root length, lettuce; (**C**) stem length, *S. lappa*; (**D**) stem length, lettuce; (**E**) fresh weight, *S. lappa*; (**F**) fresh weight, lettuce).

Seedlings of *S. lappa* and lettuce were treated with the purified compounds at different concentrations. The results demonstrated that compounds **2**, **3** and **4** inhibited the root growth of *S. lappa* significantly in a concentration-dependent manner with an inhibition rate of up to 70% and reduced to about 25% as the concentrations decreased (Figure 3A). Compounds **1**, **5**, **6** and **7** displayed no effect on the roots (Figure 3A). Similarly, compounds **2**, **4** and **7** remarkably stunted lettuce seedlings (Figure 3B). The same trends were also found in growth of stems and fresh weight (Figure 3C–F). In particular, compounds **2** and **4** inhibited the stem growth of the two plants considerably (Figure 3C,D). Additionally, it was found that roots were affected more acutely than stems. For example, inhibition of compound **2** at 200 μg/mL on roots and stems of *S. lappa* was 70% and 53%, respectively (Figure 3A,C), and similarly, compound **7** inhibited roots and stems of lettuce by 59% and 35%, respectively (Figure 3B,D).

In previous reports about allelopathy [16,17,18], crude extract from plants was usually considered to be allelochemical. However, the crude extract should not be the actual allelochemicals of the plants, because allelochemicals are compounds released by plants into the environment. Purified compounds in this work were obtained from the in-situ sampled soil and the sampling method guarantee the accuracy of the allelochemicals. Their activities to the plants were also clarified in this paper. Our findings indicated that compounds **2**, **3** and **4** affected the growth of *S. lappa*, which supplied unambiguous action media and research target in further study on the replanting obstacles in production.

It was notable that the seedling roots were more sensitive to the chemicals than the stems, which could be an indicator of the causative relationship between replanting and poor growth of roots. These compounds accumulated in soil and would change the soil components, exerting influence on the plant itself, even on the microorganisms in the soil. Thus, the measures changing the allelochemicals in the soil may affect the autotoxicity of the plant.

### 2.3. Stress Effects of the Isolated Compounds on the Protection Enzyme and ROS Accumulation in Treated Seedlings

**Figure 4:**
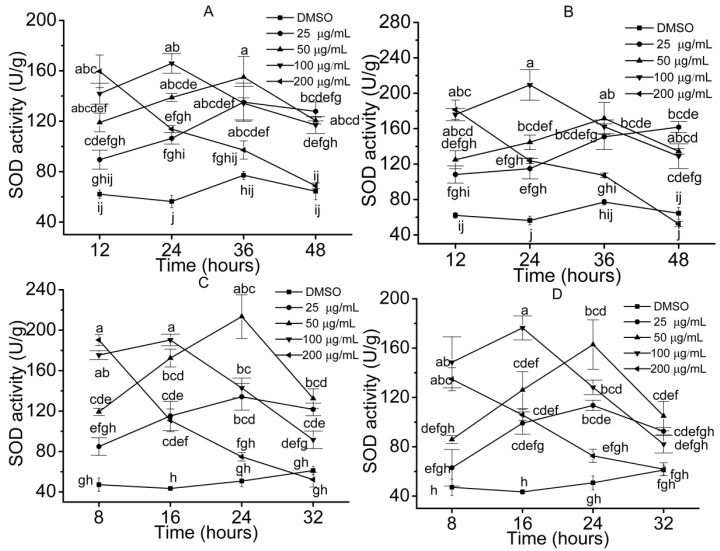
SOD activities in the seedling cells. Error bars are standard errors of the means, *n* = 3. The values were analyzed by a two-way ANOVA test, *p* = 0.05. ((**A**) *S. lappa*, compound **2**; (**B**) *S. lappa*, compound **4**; (**C**) lettuce, compound **4**; (**D**) lettuce, compound **7**).

**Figure 5:**
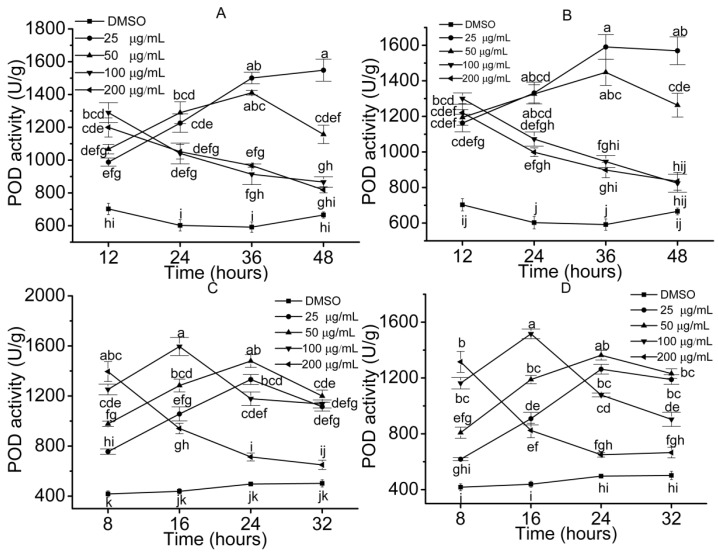
POD activities in the seedling cells. Error bars are standard errors of the means, *n* = 3. The values were analyzed by a two-way ANOVA test, *p* = 0.05. ((**A**) *S. lappa*, compound **2**; (**B**) *S. lappa*, compound **4**; (**C**) lettuce, compound **4**; (**D**) lettuce, compound **7**).

Antioxidant system, including ROS, SOD, POD and MDA were kinetically inspected to access the stress effects of the allelochemicals on the plant cells and physiological activity. The results demonstrated that SOD activities increased to the maximums and then decreased slowly over time when seedlings were treated with the compounds which showed high inhibition to growth at low concentrations (≤50 μg/mL) (Figure 4). For example, the activities of SOD in *S. lappa* and lettuce seedlings increased in initial stage, and then declined moderately when seedlings were treated with compound **4** (≤50 μg/mL). However, it showed a downtrend when the seedlings were treated with high concentrations (≥100 μg/mL) of compounds. For example, treated with 200 μg/mL of compound **4**, SOD activity of the lettuce decreased from 190 U/g to 50 U/g (Figure 4C). POD is another significant enzyme in removing excessive ROS in plant cells and behaved similarly to SOD in the treated seedlings (Figure 5). Dissimilarly, the activity of POD changed more extensively than that of SOD. For example, when treated with compound **2** at concentrations of 25 and 50 μg/mL, SOD activity in *S. lappa* seedlings increased by 51% and 26%, and correspondingly, the increase of POD was 59% and 34%, respectively.

**Figure 6:**
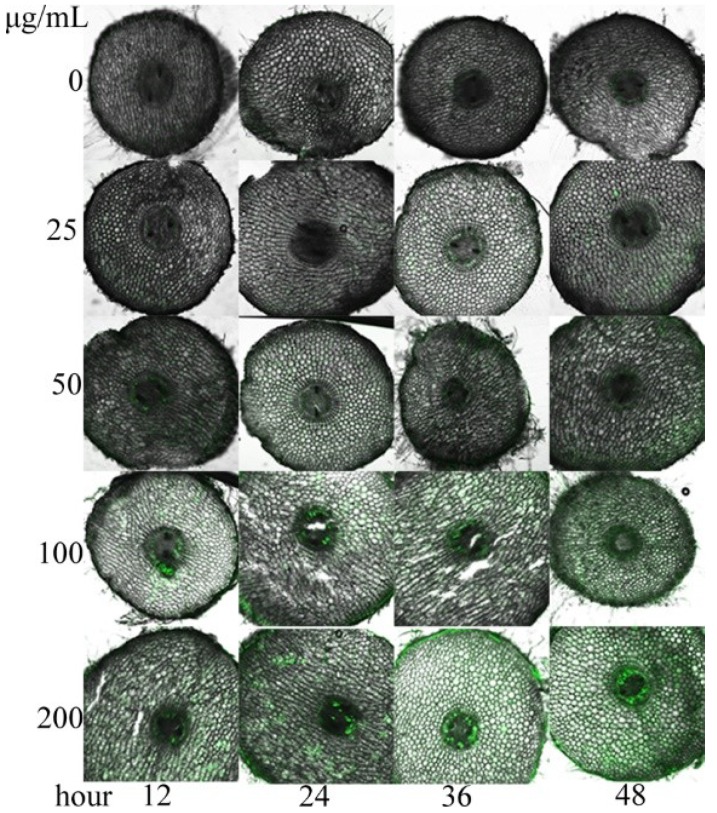
ROS in the plant tissues treated with compound **2**. Conjugates of ROS and fluorescent dye presented green fluorescence in the figure.

The ROS in the cells of treated *S. lappa* seedlings were also analysed (Figure 6) when the main antioxidant enzymes were affected by the compounds. In general, they increased with the rising concentration and action time of the compounds. When the compounds were at low concentrations, ROS moderately increased, for the probable reason that the enzyme activities were high (Figure 4 and Figure 5) and excessive ROS could be duly eliminated. Enzyme activities decreased with the rise of the compounds’ concentrations or exposure time, resulting in accumulation of ROS and peroxidation of plasma membrane in the cells. Thus, it was found that the ROS in the cells were highly correlated with the enzyme activities and cell status, and the allelochemicals may trigger systematic response of the antioxidant mechanism in cells [36].

**Figure 7:**
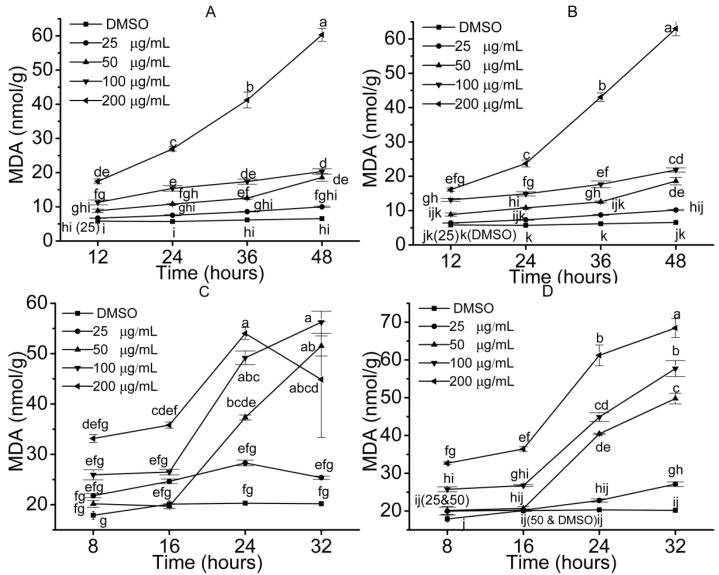
MDA concentrations in the seedling. Error bars are standard errors of the means, *n* = 3. The values were analyzed by a two-way ANOVA test, *p* = 0.05. ((**A**) *S. lappa*, compound **2**; (**B**) *S. lappa*, compound **4**; (**C**) lettuce, compound **4**; (**D**) lettuce, compound **7**).

MDA results from the peroxidation of plasma membrane caused by excessive ROS levels and reflects damage to cells. It is closely related with the enzyme activity and ROS. At low concentrations of the compounds, MDA increased slowly (Figure 7), activities of enzymes increased and ROS were low over time. As action time or concentration of the compounds increased, MDA sharply rose (Figure 7), indicating that seedlings accumulated high levels of MDA peroxidation and peroxidation of plasma membrane occurred. It was in accordance with the enzymes activities (Figure 4 and Figure 5) and levels of ROS (Figure 6).

Within the physiological responses to allelochemicals, the antioxidant system is one of the most fundamental and rapid responses taken by plants to reduce the stress from the environment. As the local and systemic secondary messenger, ROS are normal products of cell physiological activities, staying at a dynamic equilibrium between generation and removal. When the cells are over stressed, disequilibrium to the balance and ROS accumulation would be incurred.

It is definitive that allelochemicals represent an important source of stress to plants in soil, and the effects deserve to be further investigated in-depth. Besides studies on the growth of plants [37,38,39,40,41], the protective enzyme system would be a focus in further research, because it is one of the most fundamental and rapid actions taken by the plants and closely related with many critical physiological activities [16]. For example, energy supply, nutrient accumulation, immunity, material transport and biosynthesis of proteins, lipids, nucleic acids would be extremely affected when the antioxidant system is blocked [42].

Thus, allelopathy is one of the most significant factors in replanting problems, and it is probable that poor growth and plants’ diseases are associated with phytotoxicity of the allelochemicals in the soil. Moreover, as a significant result of allelopathy, the microbial community structure would also be affected by the allelochemicals, and the status of soil and microbial community structure would be deteriorated with the replanting frequencies [43,44]. It also deserves to be studied further.

### 2.4. Confirmation and Possible Release Ways of Allelochemicals

**Figure 8:**
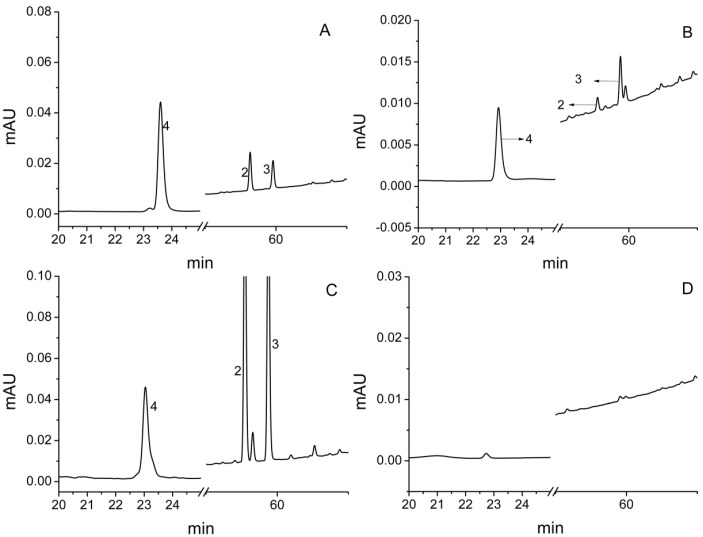
HPLC analysis of the allelochemicals in the rhizosphere soil and plant tissues of *S. lappa.* ((**A**) standard; (**B**) soil; (**C**) root; (**D**) stem and leaf).

The isolated allelochemicals of *S. lappa* were traced back to the soil by the HPLC method and their possible release pathways were also investigated. The results showed that compounds **2**, **3** and **4** existed in the rhizosphere soil (Figure 8) in amounts of 0.14, 0.76 and 0.27 μg/g, respectively (Table 1). Figure 8 indicates that they were found in the extract of the roots. Therefore, it can be assumed that compounds **2**, **3** and **4** were a part of the root exudates. Secretion and residue from the roots’ decomposition are probably the main ways allelochemicals are released into the environment [13,14,15]. The results determined the actions of allelochemicals in continuous cropping obstacle of *S. lappa* and provided some theoretical guidance for the solution of the problems.

## 3. Materials and Methods

### 3.1. General Information

^13^C- and ^1^H-NMR recorded at 100 MHz and 400 MHz were used for identifying the isolated compounds on an AM-400BB instrument (Bruker, Billerica, MA, USA). Sephadex LH-20 (25–100 mm, Sigma-Aldrich, St. Louis, MO, USA), TLC plates and silica gel (200–300 mesh, Haiyang Chemical Co. Ltd., Qingdao, China) were used for the isolation of the compounds. Spots on the TLC plates were observed under ultraviolet (UV) light and by heating after spraying with 5% (*v*/*v*) H_2_SO_4_ in C_2_H_5_OH. Reagents used in the isolation and determination were analytical and HPLC grade (Thermo Fisher, Waltham, MA, USA), respectively. A fluorescence microscope (80i, Olympus, Tokyo, Japan) was used for the detection of ROS. Biochemical reagents were purchased from Roche (Basel, Switzerland) and Sigma. A HPLC system equipped with a 2998 UV detector (DAD, Waters 2998, Milford, MA, USA) and a ODS C18 column (250 mm × 4.6 mm × 5 μm, Waters Co., Ltd.) was used for the quantification of the compounds.

### 3.2. Soil and Plant Samples

Soil surrounding the roots of *S. lappa* was collected from Wen County of Gansu Province (105.26052° E, 32.74646° N) in China on December 16, 2016. After removing the sundries and fibrous roots, the soil samples were air-dried in the dark at room temperature (about 5–10 °C) for 3 days. Then, they were ground and screened (1 mm). Sample of 2.5 kg of soil was subjected to ultrasonic extraction for 3 times by using 3 L methanol at 38 °C, for 1 h each time. *S. lappa* plant tissues, including roots, stems and leaves, were also gathered from the same field and identified by Professor Huanyang Qi from the Lanzhou Institute of Chemical Physics, Chinese Academic of Science. Fragments of plant material were initially covered with methanol for 24 h, and then ultrasonically extracted five times at 38 °C using 1 L methanol for each 200 g of the samples. The extracts were concentrated to dryness on a rotary evaporator. To investigate the autotoxicity and allelopathic activities of the chemicals in other plants, *Lactuca sativa* L. (lettuce) was also used in the experiment as a typical allelopathy-sensitive receptor plant. Seeds of *S. lappa* and lettuce were purchased from a local seed shop (Jiawang, Sichuan, China).

### 3.3. The Isolation and Identification of the Allelochemicals

Initially, silica gel column chromatography (3 cm × 40 cm) was used for the separation of the crude extract by using a step gradient elution with ether/EtOAc (80:1, 70:1, 60:1, 50:1, 40:1, 35:1, 30:1, 25:1, 20:1, 15:1, 10:1, 5:1, 3:1, 1:1, 1:3, 1:6 and 1:10, *v*/*v*) as a mobile phase followed by EtOAc/methanol (20:1, 10:1, 5:1, 2:1, 1:2, 1:5 and 1:10, *v*/*v*) and finally eluted with methanol. Guided by TLC, 20 fractions (A–T) were obtained. Preliminary bioassays showed fractions C, F, J, L, N and U inhibited the seedling growth of *S. lappa* and lettuce. These fractions were further isolated to obtain the purified compounds.

Compound **1** was isolated from fraction C by silica gel column chromatography (1 cm × 50 cm) eluting with ether/EtOAc (20:1, 15:1, 10:1 and 5:1, *v*/*v*) and purified by Sephadex LH-20 column (1 cm × 80 cm) eluting with CHCl_3_/CH_3_OH (1:1, *v*/*v*). Compound **2** was obtained from fraction F by silica gel column chromatography (1 cm × 50 cm) with a mobile phase system of ether/EtOAc, 15:1, 10:1, 8:1 and 5:1, *v*/*v* and preparative TLC. Separation of fraction J by silica gel column chromatography (1 cm × 50 cm) eluting with ether/EtOAc (10:1, 8:1, 5:1 and 3:1, *v*/*v*) and preparative TLC yielded compound **3**. Fraction L was separated by preparative TLC with ether/EtOAc (5:1) as developing solvent to obtain compound **4**. Compounds **5** and **6** were isolated from fraction N on silica gel column (1 cm × 50 cm) with ether/EtOAc, 40:1, 35:1, 30:1, 25:1, 20:1 and 15:1 mobile phases and a LH-20 column (1 cm × 80 cm) eluting with CHCl_3_/CH_3_OH (1:1). Fraction U was separated by silica gel column chromatography (1 cm × 50 cm) eluting with CHCl_3_/CH_3_OH (8:1, 6:1, 4:1, 2:1 and 1:1) followed by a LH-20 column (1 cm × 80 cm) with a CHCl_3_/CH_3_OH, 1:1 mobile phase system to obtain compound **7**. Raw data of identifications to the compounds were in the Supplementary Materials. The compounds were identified as follows:

*Methyl palmitate* (**1**): HR-ESI-MS: [M + Na]^+^, C_17_H_34_NaO_2_, measured *m*/*z* 293.2447, calculated *m*/*z* 293.2451, err 1.5 ppm. ^1^H-NMR (CDCl_3_) δ 0.88 (3H, t, 6.56Hz, H-16), 1.261–0.28 (24H, m, H: 415–), 1.62 (2H, m, H-3), 2.30 (2H, t, 7.48Hz, H-2), 3.66 (3H, s, H-17). ^13^C-NMR (CDCl_3_) δ 14.09 (C-16), 22.6934–0.12 (C: 215–), 51.39 (C-17), 174.29 (C-1).

*Costunolide* (**2**): HR-ESI-MS: [M + Na]^+^, C_15_H_20_NaO_2_, measured *m*/*z* 255.1365, calculated *m*/*z* 255.1356, err −3.9 ppm. ^1^H-NMR (CD_3_COCD_3_) δ 1.47 (3H, s, 6-CH_3_), 1.72 (3H, s, 10-CH_3_), 1.741−.81 (2H, m, H-4), 2.182–0.37 (6H, m, H-5, 8, 9), 2.66 (1H, t, 9.64Hz, H-3), 4.69 (1H, t, 9.24Hz, H-12), 4.82 (1H, d, 9.96Hz, H-11), 4.90 (1H, d, 11.28Hz, H-7), 5.61 (1H, d, 3.24Hz, 2-CH_2_a), 6.10 (1H, d, 3.56Hz, 2-CH_2_b). ^13^C-NMR (CD_3_COCD_3_) δ 15.39 (6-CH_3_), 16.47 (10-CH_3_), 25.89 (C-8), 27.68 (C-4), 39.07 (C-5), 40.80 (C-9), 50.13 (C-3), 81.34 (C-12), 118.10 (2-CH_2_), 126.59(C-7), 127.88 (C-11), 137.11 (C-6), 140.61 (C-10), 141.08 (C-2), 169.61 (C-1).

*Dehydrocostus lactone* (**3**): HR-ESI-MS: [M + Na]^+^, C_15_H_18_NaO_2_, measured *m*/*z* 253.1190, calculated *m*/*z* 253.1199, err 3.5 ppm. ^1^H-NMR (CDCl_3_) δ 1.371–0.97 (4H, m, H-4, 8), 2.122−.58 (5H, m, H-5, 7, 9), 2.842−.95 (2H, m, H-3, 11), 4.82 (1H, s, 6-CH_2_b), 4.90 (1H, s, 10-CH_2_b), 5.27 (1H, d, 1.72 Hz, 10-CH_2_a), 5.07 (1H, d, 1.56 Hz, 6-C=CH_2_a), 5.49 (1H, s, 2-CH_2_b), 6.22 (1H, s, 2-CH_2_a). ^13^C-NMR (CDCl_3_) δ 30.32 (C-8), 30.95 (C-4), 32.61 (C-9), 36.27(C-5), 45.15 (C-3), 47.64 (C-7), 52.05(C-11), 85.25 (C-12), 109.64 (10=C), 112.64 (6=C), 120.19 (2=C), 139.78 (C-2), 149.25 (C-6), 151.25 (C-10), 170.27 (C-1).

*Scopoletin* (**4**): HR-ESI-MS: [M + Na]^+^, C_10_H_8_NaO_4_, measured *m*/*z* 215.0316, calculated *m*/*z* 215.0315, err 0.8 ppm. ^1^H-NMR (CDCl_3_) δ 3.96 (3H, d, 6-CH_3_), 6.27 (1H, d, 9.48 Hz, H-2), 6.85 (1H, s, H-5), 6.92 (1H, s, H-8), 7.60 (1H, d, 9.47 Hz, H-3). ^13^C-NMR (CDCl_3_) δ 56.42 (6-OCH_3_), 103.21 (C-8), 107.50 (C-5), 111. 50 (C-4), 113.42 (C-2), 143.32 (C-3), 144.02 (C-7), 149.72 (C-6), 150.27 (C-9), 161.47 (C-1).

*Syringaldehyde* (**5**): HR-ESI-MS: [M + Na]^+^, C_9_H_10_NaO_4_, measured *m*/*z* 205.0467, calculated *m*/*z* 205.0471, err 2.0 ppm. ^1^H-NMR (CDCl_3_) δ 3.98 (6H, s, 3, 5-OCH_3_), 6.12 (2H, s, H-2, 6), 7.16 (1H, s, 4-OH), 9.82 (1H, s, CHO). ^13^C-NMR (CDCl_3_) δ 56.49 (3, 5-OCH_3_), 106.70 (C-2, 6), 128.41 (C-1), 140.83 (C-4), 147.37 (C-3, 5), 190.78 (CHO).

*5-Hydroxymethylfurfural* (**6**): HR-ESI-MS: [M + Na]^+^, C_6_H_6_NaO_3_, measured *m*/*z* 149.0208 calculated *m*/*z* 149.0209, err 0.7 ppm. ^1^H-NMR (CDCl_3_) δ 2.77 (1H, br, 5-C-OH), 4.72 (2H, s, 5-CH_2_-), 6.52 (1H, d, 2.84Hz, H-4), 7.23 (1H, d, 3.4Hz, H-3), 9.56 (1H, d, 3.84Hz, CHO).^13^C-NMR (CDCl_3_) δ 57.59 (5-C-OH), 110.02 (C-4), 122.95 (C-3), 152.35 (C-2), 160.71 (C-5), 177.74 (CHO).

*Chlorogenic acid* (**7**): HR-ESI-MS: [M + Na]^+^, C_16_H_18_NaO_9_, measured *m*/*z* 377.0844, calculated *m*/*z* 377.0843, err 0.2 ppm. ^1^H-NMR (CD_3_OD) δ 2.012–0.09 (2H, m, H-2a, 6a), 2.142–0.24 (2H, m, H-2b, 6b), 3.73 (1H, dd, 3.08, 3.08Hz, H-5), 4.16 (1H, m, H-4), 5.32 (1H, m, H-3), 6.25 (1H, d, 15.88Hz, -COCH=), 6.77 (1H, d, 8.16Hz, H-5’), 6.94 (1H, dd, 1.88, 1.88Hz, H-6’), 7.04 (1H, d, 1.84Hz, H-2’), 7.55 (1H, d, 15.92Hz, CO-C=CH). ^13^C-NMR (CD_3_OD) δ 36.81 (C-6), 37.38 (C-2), 69.90 (C-5), 70.58 (C-3), 72.07 (C-4), 74.77 (C-1), 113.80 (C-2′), 113.86(C-5′), 115.09 (-C=), 121.59 (C-6′), 126.40 (C-1′), 145.40 (=C-Ar), 145.70 (C-4′), 148.18 (C-3′), 167.28 (-CO-), 175.66 (COOH).

### 3.4. Activities of the Purified Compounds on the Growth of Seedlings

Inhibition to the seedlings was used for evaluating the bioactivity of the purified compounds. Seeds of *S. lappa* and lettuce were immersed in warm water at 37 °C for 3 h to accelerate germination, then sterilized with 5% sodium hypochlorite solution for 2 min and washed with distilled water to remove the residue of solution. The seeds were germinated on wet filter paper and kept humid in dark at 25 °C for 3 days and 24 h for *S. lappa* and lettuce, respectively. Uniformly germinated seeds were cultured in 6-well plates for further tests when their coats were just broken. Crude extract was dissolved in DMSO, diluted with water and administered on the seedlings at concentrations of 400, 200, 100 and 50 μg/mL, respectively, with three replicates for each treatment. The concentrations of DMSO were all less than 1% (*v*/*v*) in the samples, and the solution containing only the same concentration of DMSO was used in the controls. Purified compounds were used with the same method at the concentrations of 200, 100, 50 and 25 μg/mL. After being incubated in a constant-temperature humidity chamber in dark at 25 °C (*S. lappa* for 60 h, lettuce for 36 h), the root length, stem length and fresh weight of *S. lappa* and lettuce seedlings were measured. The inhibition ratios were calculated according to the following formula:

(length or weight of control −length or weight of treated)length or weight of control×100%

### 3.5. Stress Effects of Allelochemicals on Antioxidant Enzymes in Treated Seedlings

Peroxidase (POD), superoxide dismutase (SOD) and malondialdehyde (MDA) are all important physiological indexes in plant and usually used for the investigation of antioxidant activity and plasmolemma structure status. Seedling roots of *S. lappa* and lettuce were ground in cold PBS (100 mM, pH = 7) and centrifuged (10,000 rpm/min) at 4 °C. The suspensions were used for the spectrophotometric determination methods [45,46,47]. POD activities were determined by mixing 2.9 mL of PBS (0.05M, pH = 5.5), hydrogen peroxide and substrate (guaiacol) together, and then observed the absorbance at 470 nm at the interval of 30 s for 4 min [45]. The results were calculated by the formula:

Δabsorbance(0.01×fresh weight×4)

SOD activities were measured by a modified hydroxylamine method [46]. Suspensions (100 μL) containing 2-(4-iodophenyl)-3-(4-nitrophenyl)-5-(2,4-disulfophenyl)-2*H*-tetrazolium monosodium salt solution (WST-1, 5 μL, 0.05 mM in PBS), xanthine oxidase ammonium sulfate suspension (25 μL, 1.5 μ/mg of protein in PBS), catalase solution (5 μL, 10 μg/mL in PBS) and hypoxanthine solution (865 μL, 0.1 mM in PBS with 0.1 mM diethylenetriaminepentaacetic acid) were added into the reaction system. For the control groups, all the conditions were the same except the sample suspension was replaced by distilled water. The reaction system was incubated at 25 °C for 30 min and measured at 560 nm. SOD activity was calculated by the formula:

2×dilution multiples×(control absorbance−sample absorbance)control absorbance×100%

MDA concentration was tested by the thiobarbituric acid (TBA) method [47]. TBA solution (2.5 mL, 2% in 20% trifluoroacetic acid) was mixed together with the sample suspensions (1.5 mL). The reaction system was heated in water at 90 °C for 30 min and then centrifuged (12,000 rpm per min) for 20 min. The suspension was measured at 450 nm, 532 nm and 600 nm. The results were calculated by the formula:

6.45×(absorbance 532 nm−absorbance 600 nm)0.56×absorbance 450 nm

### 3.6. Effects of Allelochemicals on the Accumulation of ROS in the Treated Seedlings

ROS in the cells of treated seedlings were observed by the method in [48] with slight modifications. 2′,7′-Dichlorofluorescein diacetate (DCFH-DA), used for labeling the ROS, was dissolved in DMSO and diluted with distilled water to prepare DCFH-DA at a concentration of 20 μmol/L. The proportion of DMSO was less than 1%. Compound **2** showing high allelopathic activity was used to treat the seedlings, comparing with the controls only treated with DMSO. Freehand sections of the roots were washed with distilled water four times and labeled for 8 min in dark with DCFH-DA solution. Then the sections were washed with distilled water six times to remove the dye residue and viewed under a fluorescence microscope (excitation wavelength 488 nm, emission wavelength > 520 nm).

### 3.7. Statistical Analysis

Data was expressed as the mean ± standard error (SE). Variance analysis to the values was conducted by SPSS Statistics (version 19.0, IBM, Amunk, NY, USA). Results of inhibition to the seedling growth were subject to one-way analysis of variance (ANOVA) with a Dunnett’s test at probability levels of 0.05 and 0.01. Values of stress effects were analyzed by a two-way ANOVA test with the *p* value, 0.05.

### 3.8. Analysis for the Possible Release Ways of Allelochemicals

To investigate the enrichment location of the allelochemicals in the environment and support the measures for dealing with the replanting obstacles, the allelochemicals were traced back and their possible release pathways were studied. Extract of plant tissue and soil of *S. lappa* was resolved and filtered (0.22 μm) before analysis. Analysis of the samples was carried out on an HPLC system at 35 °C. The detection wavelength was 230 nm and the injection volume was 20 µL. Ultrapure water (A) and acetonitrile (B) were used to prepare the mobile phase as follows: linearly varying (A) from 85% to 10% in 70 min at the flow rate of 1.0 mL/min and then held for 10 mins.

## 4. Conclusions

In this study, costunolide, dehydrocostus lactone and scopoletin were obtained from the rhizosphere soil of *S. lappa*. They caused intensive inhibition on the tested seedlings and are considered to be the main allelochemicals. Their contents in rhizosphere soil and release pathways were also determined. Activities of SOD and POD in the treated seedlings evidently respond to the stress from the allelochemicals, and ROS exhibited an accumulation trend. The increase of MDA indicated that the plasma membrane was damaged by the accumulated ROS. The results suggested that the allelochemicals stressed the receptor plant, and this caused a disequilibrium in the self-protection mechanism of the cells and damaged the cell structure. The allelochemicals affected the growth and the antioxidant enzymes of the seedlings, which would explain the obstacles to many physiological activities in the growth of the plant, and it might be an initial inducement to replanting obstacles. It also might provide a clue worth exploring to help mitigate replanting problems of *S. lappa*.

## Figures and Tables

**Table 1:** Qualitative and quantitative analysis of the allelochemicals in the soil.

Compound	Retention Time (RT, min)	Concentration (μg/g)
**2**	58.18	0.14
**3**	59.80	0.76
**4**	23.60	0.27

## Supplementary Materials

The following are available online. HR-MS and NMR spectra for compounds **1**–**7**.
