# Supplementary material for: Main Allelochemicals from the Rhizosphere Soil of Saussurea lappa (Decne.) Sch. Bip. and Their Effects on Plants’ Antioxidase Systems
Source: Molecules. 2018 Sep 30;23(10):2506. doi: 10.3390/molecules23102506 (PMC6222321; doi:10.3390/molecules23102506)
Supplement: Supplementary file 1 [file molecules-23-02506-s001.pdf]

Supplementary Materials

^{1}H NMR spectrum of compound 1

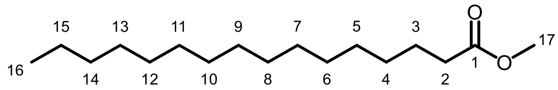

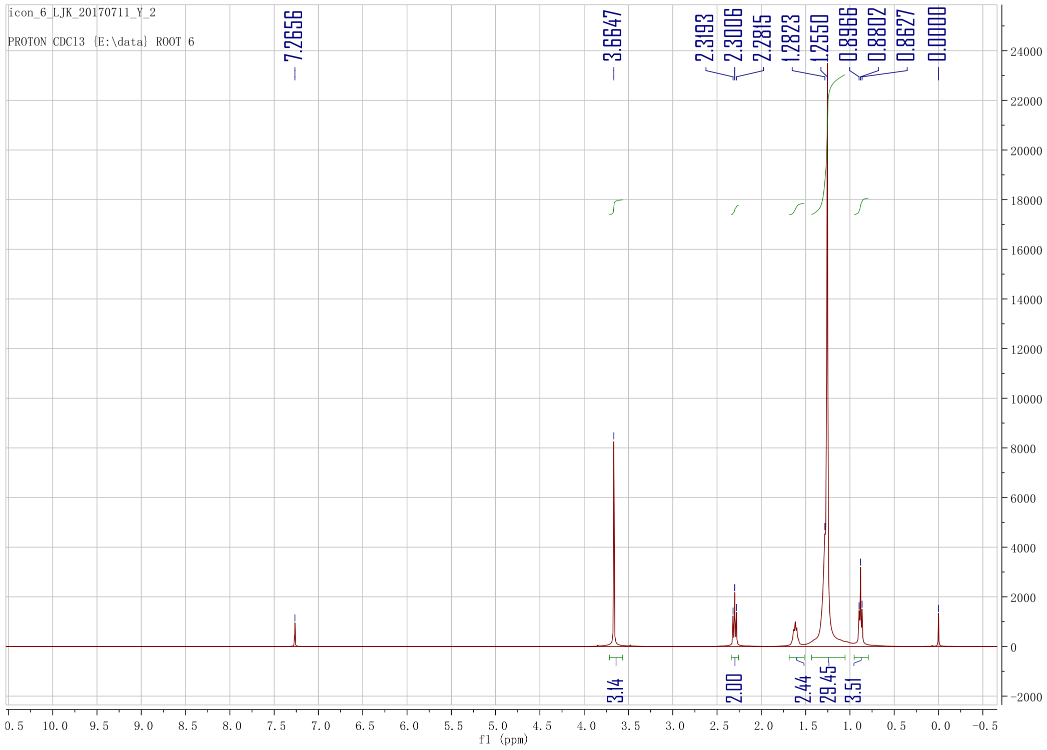

^{13}C NMR spectrum of compound 1

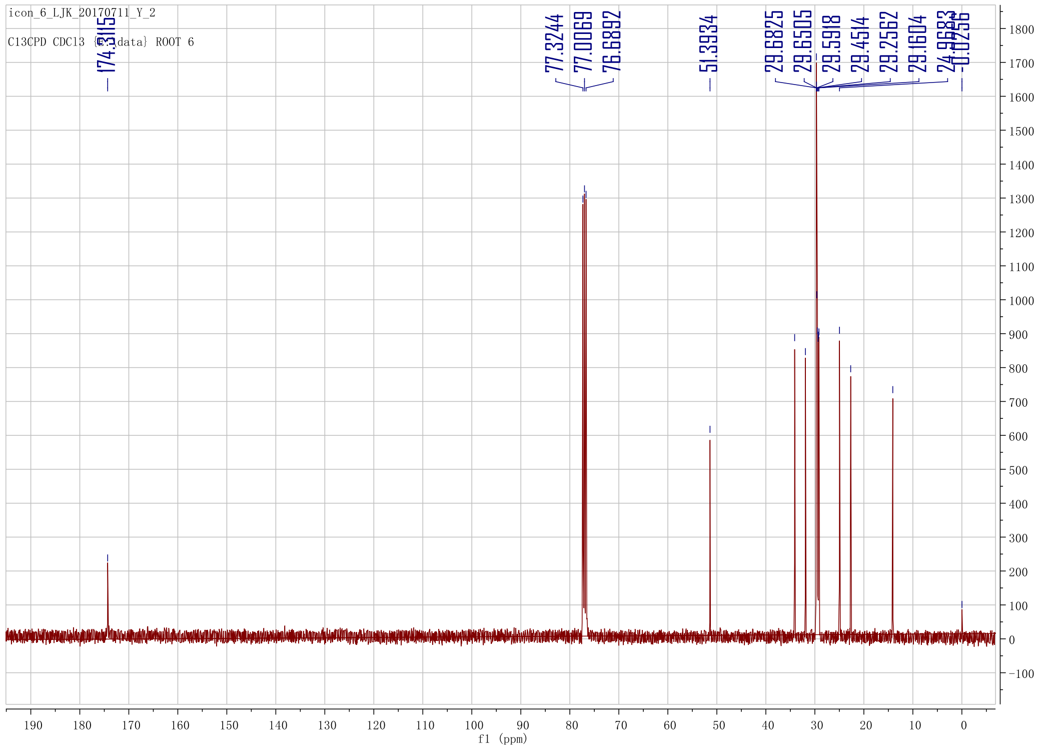

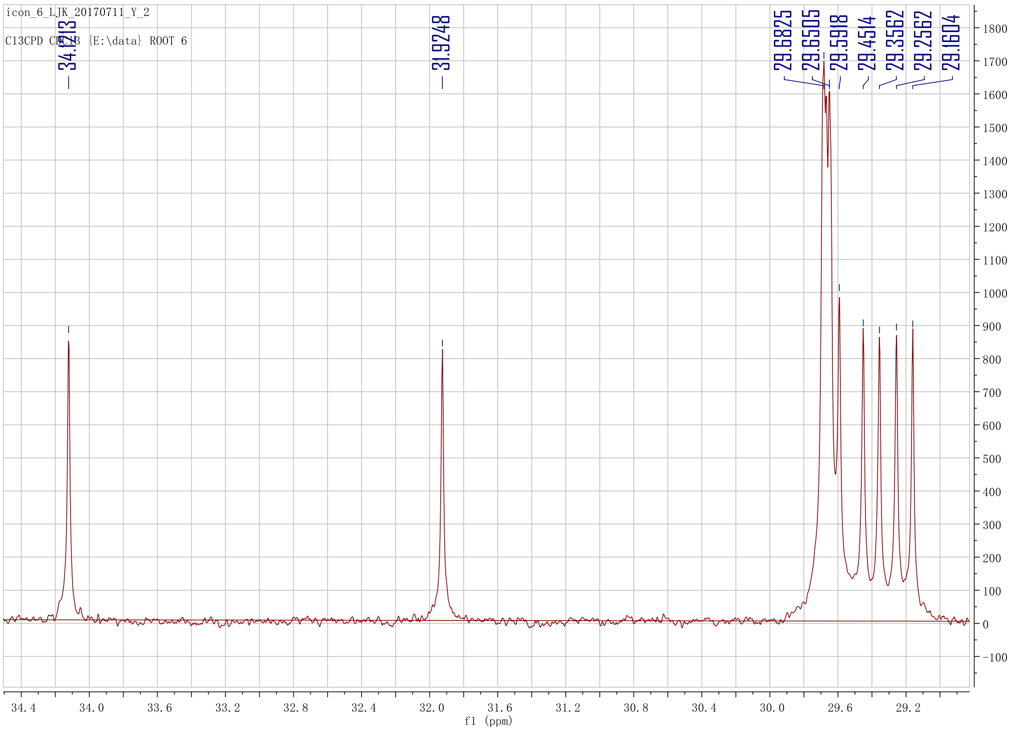

Mass spectrum of compound 1

Mass Spectrum SmartFormula Report

Analysis Info

Analysis Name	D:\Data\user\liujinkun20170815-4.d	Acquisition Date	2017-8-16 15:26:17
Method	tune_low.m	Operator	BDAL@DE
Sample Name	4	Instrument / Ser#	micrOTOF-Q 20453
Comment	compound 1		

Acquisition Parameter

Source Type	ESI	Ion Polarity	Positive	Set Nebulizer	0.4 Bar
Focus	Active	Set Capillary	4500 V	Set Dry Heater	180 ¡ãC
Scan Begin	100 m/z	Set End Plate Offset	-500 V	Set Dry Gas	4.0 l/min
Scan End	1000 m/z	Set Collision Cell RF	300.0 Vpp	Set Divert Valve	Waste

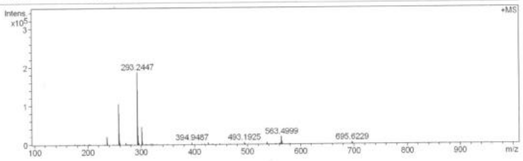

Meas. m/z	#	Formula	m/z	err [ppm]	Mean err [ppm]	rdb	N-Rule	e¡¥ Conf	mSigma	Std I	Std Mean m/z	Std I VarNorm	Std m/z Diff	Std Comb Dev
293.2447	1	C 17 H 34 Na O 2	293.2451	1.5	1.4	0.5	ok	even	13.6	20.9	0.4	15.1	0.1	842.7

Bruker Compass DataAnalysis 4.0 printed: 2017-8-16 16:35:27 Page 1 of 1

^{1}H NMR spectrum of compound 2

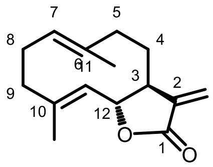

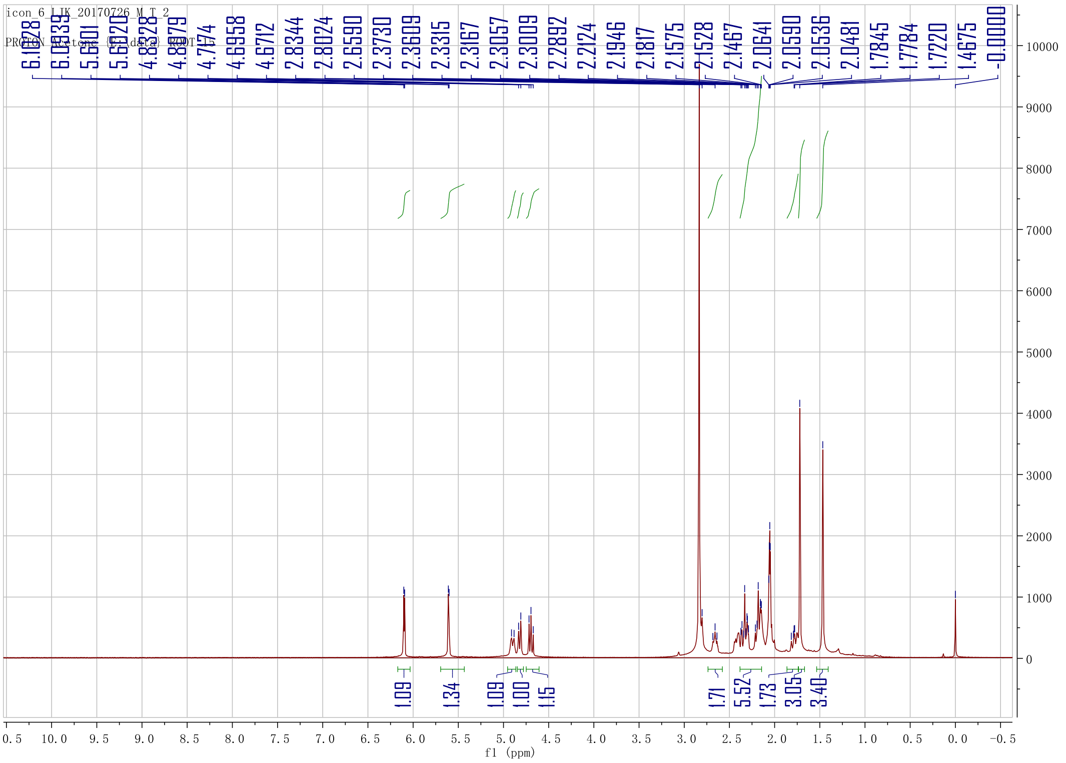

^{13}C NMR spectrum of compound 2

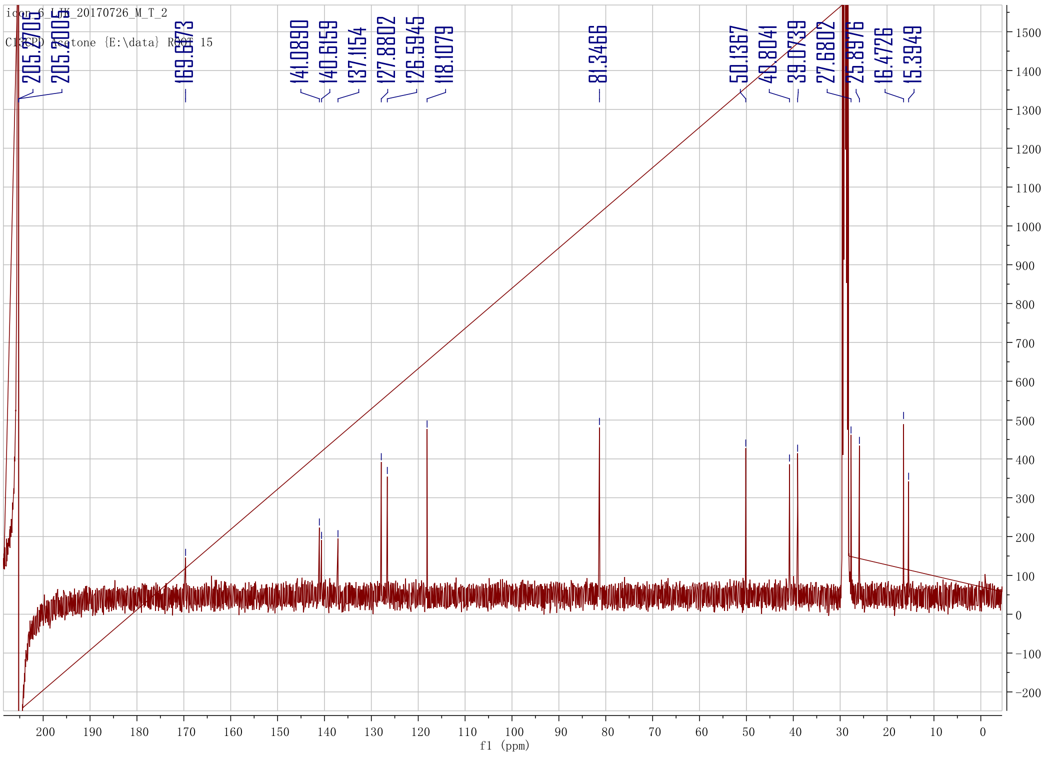

Mass spectrum of compound 2

Mass Spectrum SmartFormula Report

Analysis Info

Analysis Name	D:\Data\user\linjinkun20170816-2.d	Acquisition Date	2017-8-17 9:41:04
Method	tune_low.m	Operator	BDAL@DE
Sample Name	8	Instrument / Ser#	micrOTOF-Q 20453
Comment			

compound 2

Acquisition Parameter

Source Type	ESI	Ion Polarity	Positive	Set Nebulizer	0.4 Bar
Focus	Active	Set Capillary	4500 V	Set Dry Heater	180 ¡ãC
Scan Begin	100 m/z	Set End Plate Offset	-500 V	Set Dry Gas	4.0 l/min
Scan End	1000 m/z	Set Collision Cell RF	300.0 Vpp	Set Divert Valve	Waste

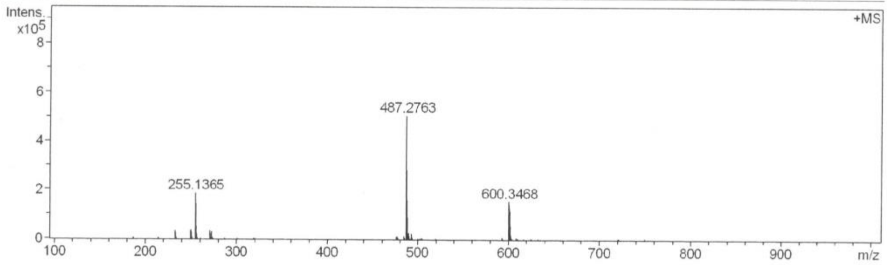

Meas. m/z	#	Formula	m/z	err [ppm]	Mean err [ppm]	rdb	N-Rule	e¡¥ Conf	mSigma	Std I	Std Mean m/z	Std I VarNorm	Std m/z Diff	Std Comb Dev
255.1365	1	C 15 H 20 Na O 2	255.1356	-3.9	-3.0	5.5	ok	even	12.0	19.5	1.0	12.8	1.8	842.7

^{1}H NMR spectrum of compound 3

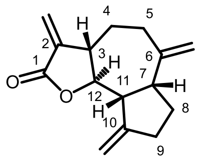

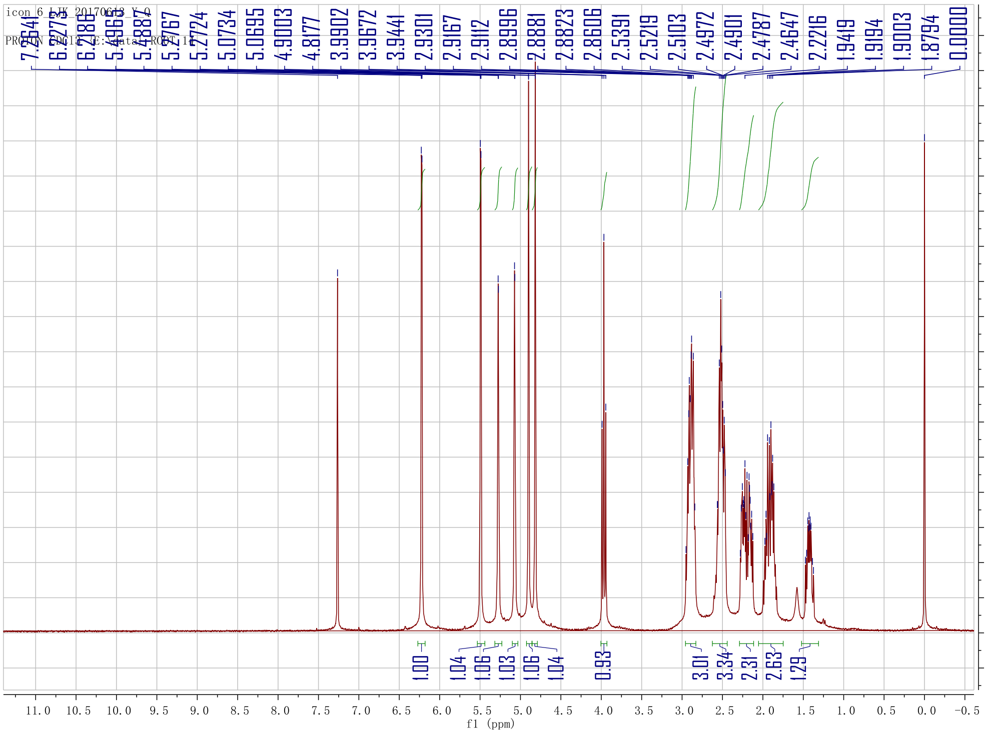

^{13}C NMR spectrum of compound 3

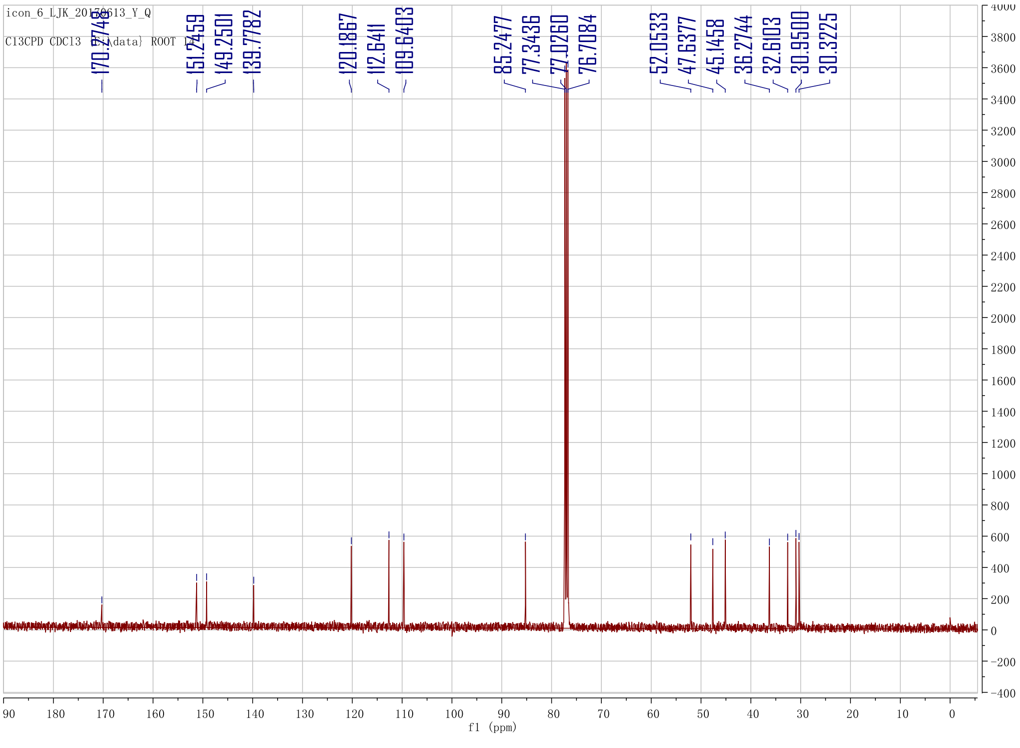

Mass spectrum of compound 3

Mass Spectrum SmartFormula Report

Analysis Info

Analysis Name	D:\Data\user\liujinkun20170815-11.d	Acquisition Date	2017-8-16 15:39:34
Method	tune_low.m	Operator	BDAL@DE
Sample Name	11 compound 3	Instrument / Ser#	micrOTOF-Q 20453
Comment			

Acquisition Parameter

Source Type	ESI	Ion Polarity	Positive	Set Nebulizer	0.4 Bar
Focus	Active	Set Capillary	4500 V	Set Dry Heater	180 ¡ãC
Scan Begin	100 m/z	Set End Plate Offset	-500 V	Set Dry Gas	4.0 l/min
Scan End	1000 m/z	Set Collision Cell RF	300.0 Vpp	Set Divert Valve	Waste

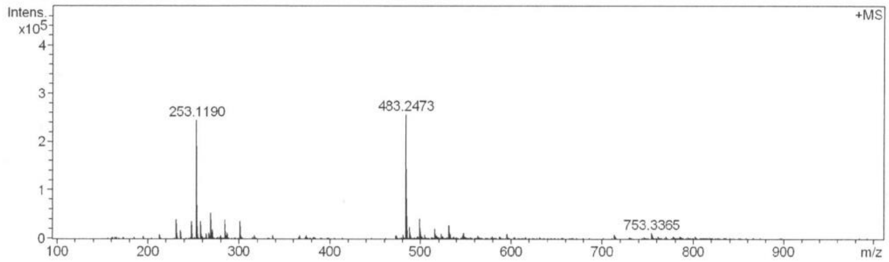

Meas. m/z	#	Formula	m/z	err [ppm]	Mean err [ppm]	rdb	N-Rule	e¡¥ Conf	mSigma	Std I	Std Mean m/z	Std I Var Norm	Std m/z Diff	Std Comb Dev
253.1190	1	C 15 H 18 Na O 2	253.1199	3.5	3.7	6.5	ok	even	17.3	30.3	1.0	15.9	0.4	842.7

^{1}H NMR spectrum of compound 4

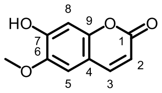

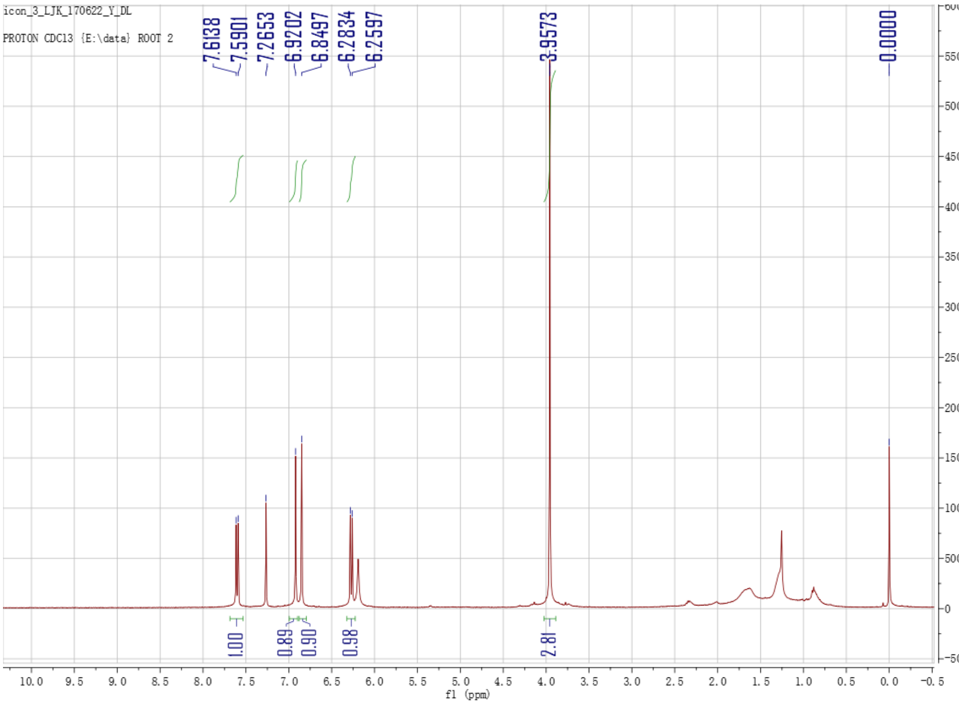

^{13}C NMR spectrum of compound 4

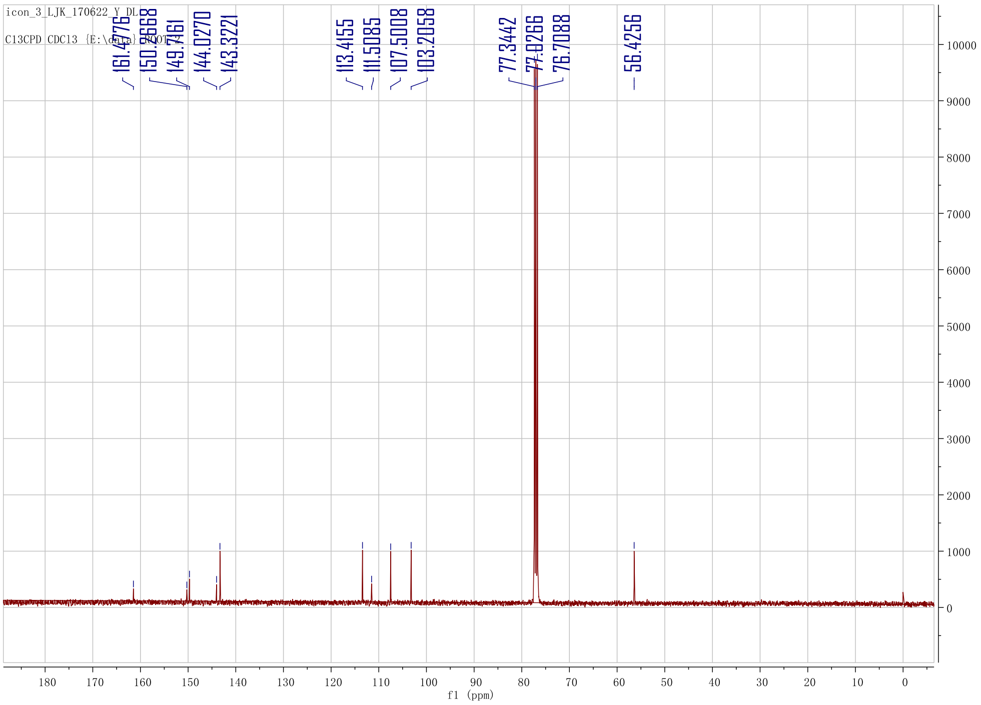

Mass spectrum of compound 4

Mass Spectrum SmartFormula Report

Analysis Info

Analysis Name	D:\Data\user\liujinkun20170815-10.d	Acquisition Date	2017-8-16 15:38:17
Method	tune_low.m	Operator	BDAL@DE
Sample Name	10 compound 4	Instrument / Ser#	micrOTOF-Q 20453
Comment			

Acquisition Parameter

Source Type	ESI	Ion Polarity	Positive	Set Nebulizer	0.4 Bar
Focus	Active	Set Capillary	4500 V	Set Dry Heater	180 ¡ãC
Scan Begin	100 m/z	Set End Plate Offset	-500 V	Set Dry Gas	4.0 l/min
Scan End	1000 m/z	Set Collision Cell RF	200.0 Vpp	Set Divert Valve	Waste

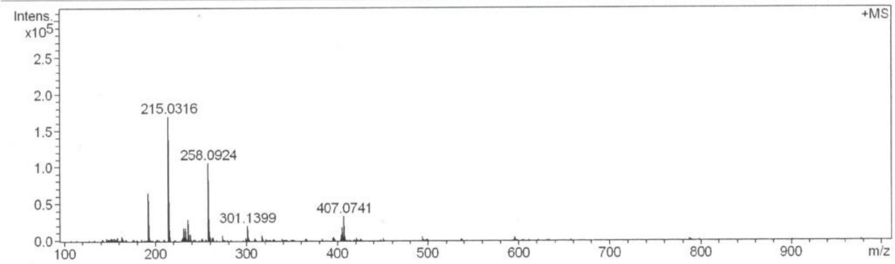

Meas. m/z	#	Formula	m/z	err [ppm]	Mean err [ppm]	rdb	N-Rule	e¡¥ Conf	mSigma	Std I	Std Mean m/z	Std I Var Norm	Std m/z Diff	Std Comb Dev
215.0316	1	C 10 H 8 Na O 4	215.0315	-0.8	-0.3	6.5	ok	even	8.1	12.8	0.3	9.9	1.1	842.7

^{1}H NMR spectrum of compound 5

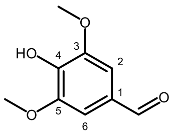

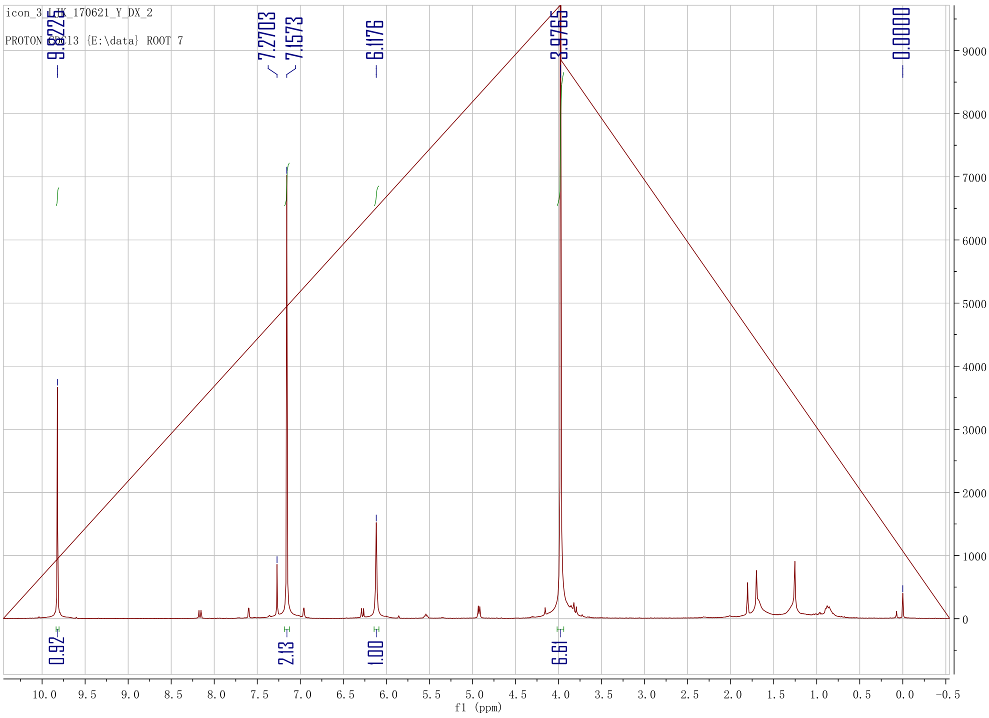

^{3}C NMR spectrum of compound 5

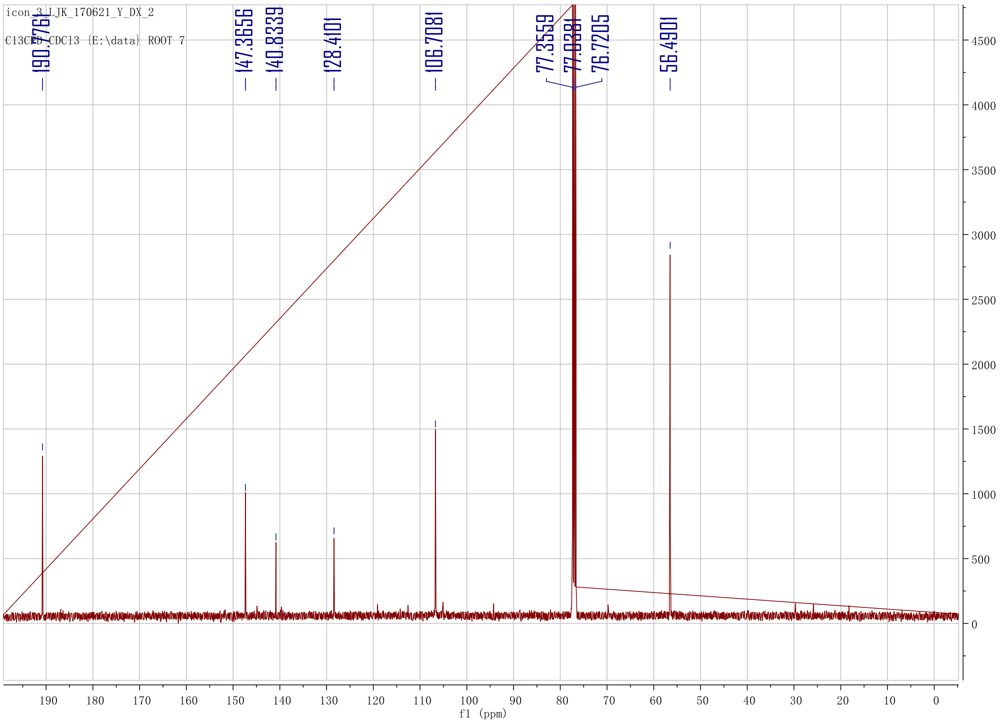

Mass spectrum of compound 5

Mass Spectrum SmartFormula Report

Analysis Info

Analysis Name	D:\Data\user\liujinkun20170815-9.d	Acquisition Date	2017-8-16 15:37:03
Method	tune_low.m	Operator	BDAL@DE
Sample Name	9 Compound 5	Instrument / Ser#	micrOTOF-Q 20453
Comment			

Acquisition Parameter

Source Type	ESI	Ion Polarity	Positive	Set Nebulizer	0.4 Bar
Focus	Active	Set Capillary	4500 V	Set Dry Heater	180 ¡ãC
Scan Begin	100 m/z	Set End Plate Offset	-500 V	Set Dry Gas	4.0 l/min
Scan End	1000 m/z	Set Collision Cell RF	200.0 Vpp	Set Divert Valve	Waste

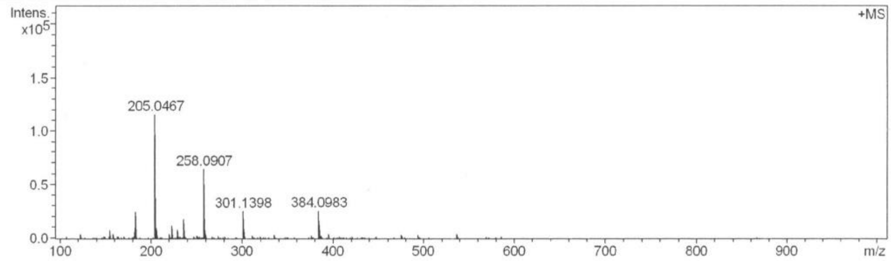

Meas. m/z	#	Formula	m/z	err [ppm]	Mean err [ppm]	rdb	N-Rule	e¡¥ Conf	mSigma	Std I	Std Mean m/z	Std I Var Norm	Std m/z Diff	Std Comb Dev
205.0467	1	C 9 H 10 Na O 4	205.0471	2.0	2.2	4.5	ok	even	10.1	18.5	0.5	11.0	0.6	842.7

^{1}H NMR spectrum of compound 6

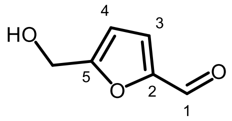

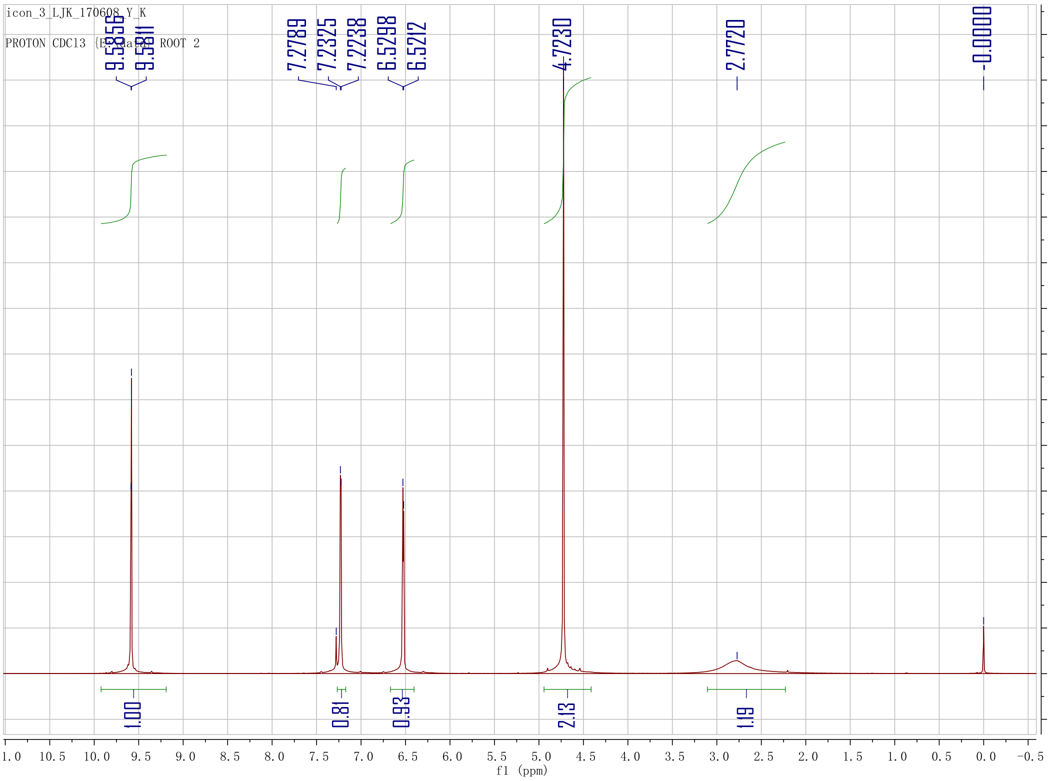

^{13}C NMR spectrum of compound 6

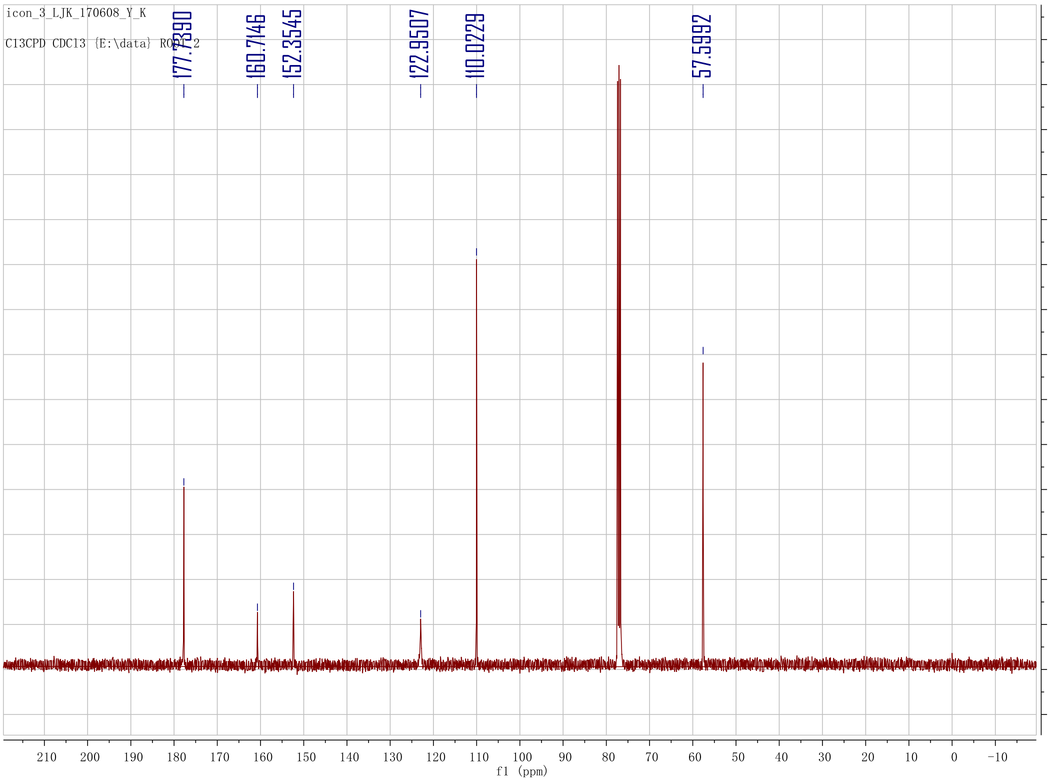

Mass spectrum of compound 6

Mass Spectrum SmartFormula Report

Analysis Info

Analysis Name	D:\Data\user\liujinkun20170815-6.d	Acquisition Date	2017-8-16 15:31:38
Method	tune_low.m	Operator	BDAL@DE
Sample Name	6 Compound 6	Instrument / Ser#	micrOTOF-Q 20453
Comment			

Acquisition Parameter

Source Type	ESI	Ion Polarity	Positive	Set Nebulizer	0.4 Bar
Focus	Active	Set Capillary	4500 V	Set Dry Heater	180 ¡ãC
Scan Begin	100 m/z	Set End Plate Offset	-500 V	Set Dry Gas	4.0 l/min
Scan End	1000 m/z	Set Collision Cell RF	150.0 Vpp	Set Divert Valve	Waste

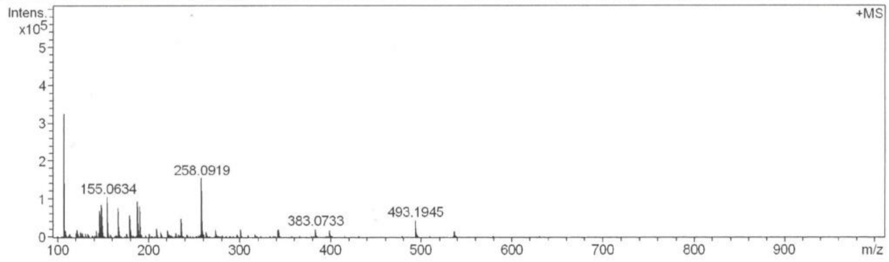

Meas. m/z	#	Formula	m/z	err [ppm]	Mean err [ppm]	rdb	N-Rule	e¡¥ Conf	mSigma	Std I	Std Mean m/z	Std I Var Norm	Std m/z Diff	Std Comb Dev
149.0208	1	C 6 H 6 Na O 3	149.0209	0.7	0.7	3.5	ok	even	37.6	65.2	0.1	37.4	0.1	842.7

^{1}H NMR spectrum of compound 7

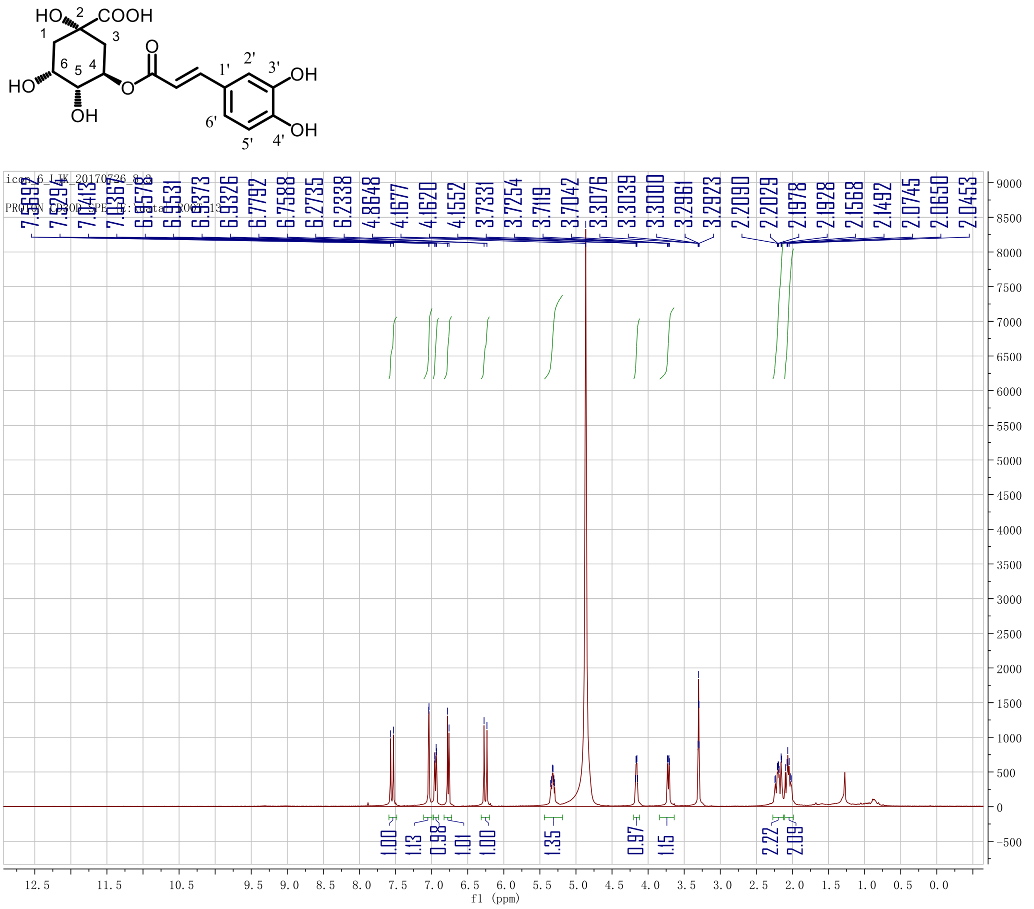

^{13}C NMR spectrum of compound 7

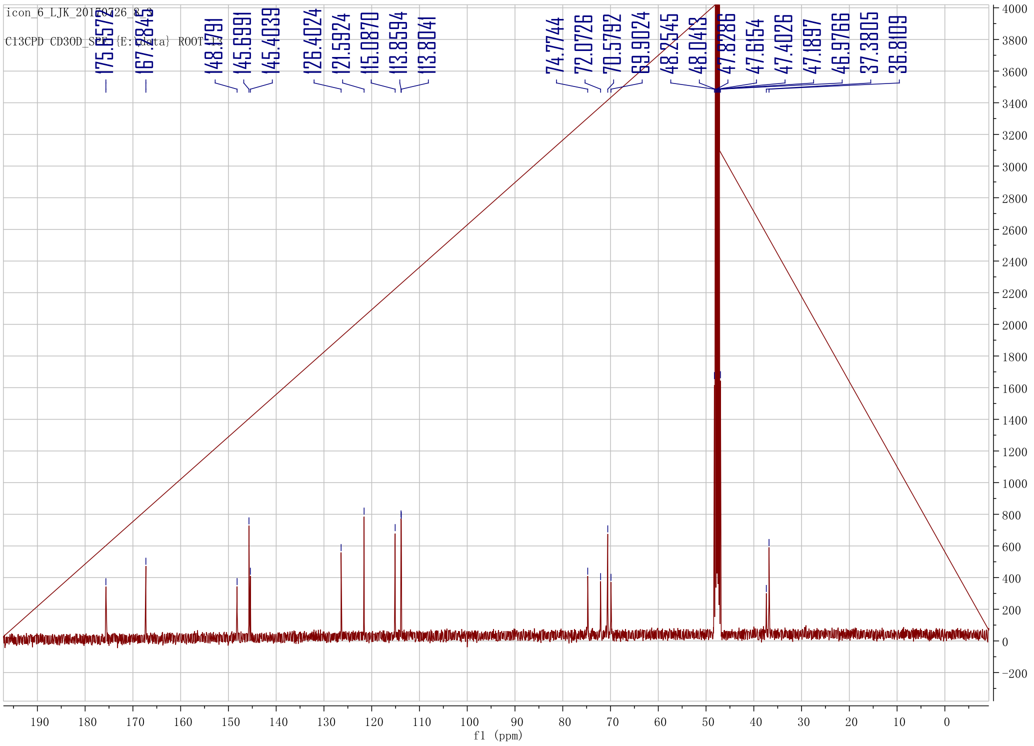

Mass spectrum of compound 7

Mass Spectrum SmartFormula Report

Analysis Info

Analysis Name	D:\Data\user\liujinkun20170815-7.d	Acquisition Date	2017-8-16 15:32:47
Method	tune_low.m	Operator	BDAL@DE
Sample Name	7	Instrument / Ser#	micrOTOF-Q 20453
Comment			

Compound 7

Acquisition Parameter

Source Type	ESI	Ion Polarity	Positive	Set Nebulizer	0.4 Bar
Focus	Active	Set Capillary	4500 V	Set Dry Heater	180 ¡ãC
Scan Begin	100 m/z	Set End Plate Offset	-500 V	Set Dry Gas	4.0 l/min
Scan End	1000 m/z	Set Collision Cell RF	300.0 Vpp	Set Divert Valve	Waste

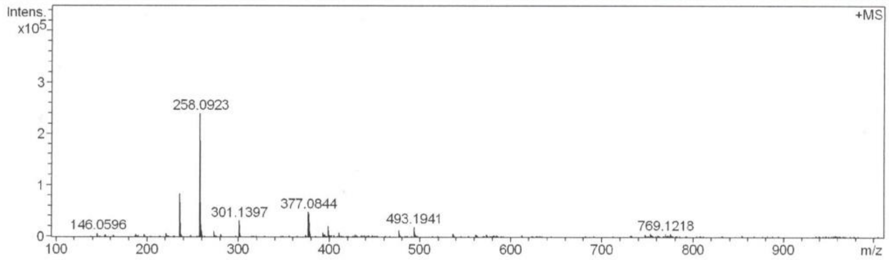

Meas. m/z	#	Formula	m/z	err [ppm]	Mean err [ppm]	rdb	N-Rule	e¡¥ Conf	mSigma	Std I	Std Mean m/z	Std I Var Norm	Std m/z Diff	Std Comb Dev
377.0844	1	C 16 H 18 Na O 9	377.0843	-0.2	0.2	7.5	ok	even	33.4	54.7	0.4	30.0	0.9	842.7
